# H.R.H. the Prince of Wales in the Chair

**Published:** 1919-12-27

**Authors:** 


### THE LEAGUE OF MERCY.

### H.R.H. the Prince of Wales in the Chair.

The twentieth annual meeting of the Presidents of the League of Mercy was held at St. James's Palace on Wednesday, December 17. The chair was occupied by the new Grand President, H.R.H. the Prince of Wales, K.G., and supporting him were H.R.H. the Duchess of Albany, H.H. Princess Helena Victoria, Viscount and Viscountess Farquhar, Lady Macclesfield, Sir Chas. Jessel, J.P., D.L., Lord Wolverton, the Dowager Lady Dimsdale, Sir Charles Wakefield, Sir Fredk. Green, Sir Wm. and Lady Phillips, Lady Tree, Sir William and Lady Collins, and the Hon. Mrs. Lawson-Johnston.

The minutes of the last meeting have been read by Sir Jas. Harrison, and signed by the Grand President.

**Col. R. J. Blackham, C.B., D.S O.,** moved : "That the Rt. Hon. the Viscount Farquhar, G.C.V.O., be appointed Chairman, Sir Fredk. Green, K.B.E., Deputy Chairman, and Mr. Arthur H. Greening Hon. Secretary, respectively." He said Lord Farquhar had been the life and soul of the League of Mercy since its inception, and a benefactor through all the years of its existence to its majority. Mr. Greening also was quite indispensable.

**Lady Tree** seconded, in a few felicitous words of commendation, and it was carried.

**Rev. E. G. Gordon** proposed : "That her Highness Princess Marie Louise, the Right Hon. the Viscount Farquhar, G.C.V.O., the Viscount Hill, Col. the Right Hon. the Lord Lambourne, C.V.O., the Hon. Mrs. George Lawson-Johnston, the Dowager Lady Dimsdale, O.B.E., and the Rev. W. H. Hornby Steer, M.A., be appointed members of the Executive Council."

He said the ladies and gentlemen enumerated, with one exception, had done such excellent work for the League that there was little need to enlarge upon it; and, as in the case of other organisations, its good work had been mainly due to its Executive Committee. It was very gratifying, and noteworthy that the Princess Marie Louise was to be added to the Council, when regard was paid to the great interest and splendid work of her Highness in connection with philanthropic bodies in all parts of London. The League would extend its warm welcome to her Highness.

**Mrs. Rodwell** seconded the resolution, and it was unanimously approved.

The Prince then handed the Bar of the Order and the Order of Mercy to the ladies and gentlemen to whom it had been respectively awarded on the approval of his Majesty as the Sovereign of the Order.

### THE PRINCE'S SPEECH.

**H.R.H. the Prince of Wales** said : Your Royal Highness, my lords, ladies and gentlemen,—I have, first of all, to read a letter from his Majesty the King, addressed to myself :

"I congratulate the Presidents, Vice-Presidents, the members, and all the workers of the League of Mercy that this year the League attains its majority, and that its active usefulness on behalf of the voluntary hospitals in and around London has been worthily maintained. (Hear, hear.) I feel certain that, under your Grand Presidency, those who have laboured so zealously to ensure that successful organisation which was aimed at by King Edward, its Founder, will continue their support to you, and that fresh recruits will be found to promote the good cause, in which the Queen and I, now as ever, take the liveliest interest." (Applause.)

I am sure that, as I do, you will all regret the absence of the Lady Grand President, Lady Athlone (hear, hear); and I am also sorry to think that Lord Athlone is not here. He has been Grand President for the last nine years, and has been a very energetic and efficient one.

Now it is a great privilege for me to take over the position of Grand President of the League of Mercy, which was founded by my grandfather, and was held in succession by my father. I am afraid that this is the first time that I have officiated or done any duty in this post.

In the first place, I offer my sincerest congratulations to the recipients of the Bar to the Order, which I have just been very pleased to present. I know that the League of Mercy was intended to enlist the sympathy and the support of small subscribers in the beneficent work of the voluntary hospitals, and to be an auxiliary to the King's Hospital Fund for London, of which I have the honour to be President. Some thousands have been so enlisted, and the activities of the League have amply justified its inauguration and vindicated the public confidence in our voluntary hospitals. I cordially congratulate all who have laboured for the League on having collected and distributed, in the last twenty years, more than £300,000. (Applause.) No one quite knew, a few years ago, what was to be the future of voluntary hospitals under a system of National Insurance, but that uncertainty has been dispelled, for the popularity of, and resort to, hospitals are as great as, and even greater than, ever. It is not improbable that the establishment of a Ministry of Health will similarly serve, not to weaken, but to strengthen confidence in these institutions. During the war, the London voluntary hospitals treated more than 100,000 of our wounded men from all theatres of war. (Applause.) At the same time this laid a great strain on all hospitals, because they were still continuing to attend to the needs of the civil population. I had myself many opportunities—on the Western Front, in Egypt, and in Italy—(applause)—of seeing the splendid services and the untiring devotion which were rendered to our wounded and sick by the doctors and the nurses who had received their training in the wards of our London hospitals. It is impossible to over-estimate the value of that training, and it is dreadful to think what might have happened had not the voluntary hospitals come to the assistance of the medical services of both the Navy and the Army during the terrible war which we have just come through. Instead of the State, as some anticipated, assuming control of the voluntary hospitals, the voluntary hospitals have, during the war, shared the responsibilities of the State. (Applause.) Accordingly it is fitting and gratifying to find that the total receipts of the League of Mercy have shown a steady rise each year during the war, and this year are—thanks largely to the generous donations of Sir Frederick Green, who has given £10,000—(applause)—and Colonel Hamilton, who has given £1,000—(applause)—and the proceeds of the Melba Matinee which Lord Farquhar held at his house, and which brought in another £1,000; also the restaurant collection of £916—so that this year the total receipts are higher than ever before. (Applause.) The grant to the King's Hospital Fund, which was £14,000 in the year before the war,

**The League of Mercy (Prince's Speech)**—(*continued*). was £16,000 last year, and Sir William Collins handed to me yesterday, as President of the King's Hospital Fund, the splendid sum of £17,000 on behalf of the League of Mercy. (Applause.) May I congratulate the League on this most gratifying sum, and on the splendid work they have done throughout the war? (Loud applause.)

**Sir Frederick Green** gave a brief sketch of the outstanding features of the League's activities during the year under review, with a few thoughts in relation to its future work. When Lord Athlone retired from the League's Grand Presidency he wrote to the Executive a letter, in which he said: "I hope that when you welcome the new President you will be able to welcome him with a bumper year." As the Prince had just said, they had been able to do that, and the League was exceedingly glad to put before His Royal Highness such a good return in this his first year of Grand Presidency.

He proceeded to point out the salient features of the year's collections. There were, he said, several very successful entertainments held during the year; indeed, all the entertainments held to benefit the League's funds were more or less successful, most of them more rather than less. A very prominent one was the splendid concert which Lord and Lady Farquhar gave at their house in Grosvenor Square. It was such a success that Lord Farquhar was able to hand over to the League even more than the Prince had stated—namely, over £1,800. No doubt a considerable part of that success was attributable to the fact that their gracious Majesties the Queen and Queen Alexandra had signified their intention to be present. Dame Melba arranged the concert, and herself sang, and the event was one of very high class. Another gratifying feature of the year's work was the restaurant collection scheme, originated and admirably worked by Colonel Bennett Dampier and an influential committee. Ladies visited large restaurants armed with collecting-boxes, and the result of their persuasive gift was that they carried away nearly £1,000. That amount was divided among the different districts, and so helped to swell the grand total.

At the head of the collections this year stood West Marylebone, with a total of £2,360. (Applause.) This was under the control of Lord and Lady Farquhar, and the sum included the proceeds of the Melba concert. South Kensington came next, with £1,602. He announced with pleasure that Princess Marie Louise had consented to become Lady President of this district. A ball at Princes Restaurant produced £130, and a sale of work, organised by Miss Mary Hart, raised over £100. The third on the list was South Paddington, with a total of £1,050. This was one of the districts which, under Lady Dimsdale, adhered to the original plan of relying upon collections without the supplementary aid of entertainments. Berkshire, under H.H. Princess Helena Victoria, realised £1,000; Epsom and Esher, £886; Fulham, £647. Mrs. Harrison had long worked in the Epsom district. She had now gone abroad; but the League welcomed the new President, lately returned from the war, Colonel Walker. There were further districts which he would like to mention, as they had risen to the occasion and tried to face what promised, a year ago, to be quite a serious situation. East Sussex achieved its record of £555, an increase of £70; East Marylebone, with £531, showed an increase of £117; Hammersmith £418, £79 increase; and South St. Pancras £354, or £37 better than last year. Ealing and Watford likewise revealed notable rises in their collections.

This year constituted a record—namely, £32,476, the previous highest having been £22,615 in 1908. It was a source of great satisfaction to him that this record should have been achieved in the year that the Prince of Wales took up his position of Grand President for the first time.

Since the last meeting the League had lost by death one of its most valued Presidents, Lord Brassey, and in the name of the gathering he tendered to Lady Brassey sincere condolences on her bereavement. He regretted, too, that Mr. Franklin had been obliged, by ill-health, to retire from the secretaryship. In his stead Miss A. E. Milnes had been appointed, and, so far, she had proved a quite satisfactory occupant of the position. Captain H. Andrews had taken up the position of organising secretary, and if any branches or districts needed assistance in the way of speaking at meetings or arranging them he would be pleased to travel down to do his best.

As to the future, he believed the coming year was going to be quite a difficult one. During the last few years things had been carried on at very high pressure. Fortunately, the actual war strain had passed, but in some respects it seemed as if war's aftermath was going to be as difficult as the actual war period. High prices were the order of the day, and these affected both victor and vanquished. They were brought about by inflated currency and the abnormal rates of exchange, with, in some years, a very poor production. Our hospitals could not be carried on under present circumstances; some of these institutions had already applied for some sort of State assistance. As the Grand President had said, those in the League believed in the voluntary system. When King Edward founded the League he wrote a letter—which was recently published in a newspaper—in which he said he thought the public had acted wisely in wishing to continue the hospitals under the voluntary system, but to do that meant that their finances must be put into a thoroughly strong position. In thinking of means to meet the difficulties of the coming year, he felt there was one class the members of which were now in better circumstances than ever before—namely, the workers; and, what was as important, they had also more leisure. A difficulty in the League had been to find time to visit the different houses to make the collections, and one of the objects of the League was to give the working man an opportunity of working with the League officials; certainly they could be of very great assistance in getting in collections. Much of the feeling of unrest evident was, he thought, due to the fact that the different classes did not know each other. Yet, from what he had seen, he believed the working man to be not only a cheerful individual, but also a good giver. If a large number of workers could be persuaded to contribute sixpence a week, it would mean a very material addition to the funds in the course of the year. He urged the League's workers to get more into touch with the humble folk in their districts. They had a duty to perform and a tradition to maintain, and he urged even increased effort to commemorate His Royal Highness's first year of Grand Presidency.

### Thanks to the Grand President.

**Viscount Farquhar** proposed a resolution of grateful thanks to H.R.H. the Prince of Wales for having presided over the meeting with such ability and charm. Wherever the Prince went he strengthened the ties of loyalty and devotion to the Throne. (Applause.)

**Colonel Sir Charles Wakefield** seconded, expressing the warm welcome felt by all at having the Prince among them to continue the gracious interest of his Royal father and mother. And those present welcomed him for his

**The League of Mercy**—(*continued*).

own sake. He was a princely ambassador, in very truth a prince of ambassadors. (Loud applause.) In his recent tour he had rendered signal service, not alone to the Empire, but to the world. The future of the world peace, the progress of humanity, was in the hands of the English-speaking peoples. The Prince's visit to the United States had been rightly hailed as a symbol of our whole-hearted desire for the closest possible friendship with the American people, and the enthusiasm with which the Prince was acclaimed wherever he went assured us that these sentiments were re-echoed in American hearts. It was an especial pleasure to welcome the Prince after the noble part he had played in moulding the destinies of a nation, thus helping to a true appreciation on the other side of the British ideal. (Applause.)

The resolution was carried by acclamation.

**The Grand President** in response, said: Your Royal Highness, my lords, ladies and gentlemen, I am very grateful and appreciative that Lord Farquhar should have moved this vote of thanks, and that Sir Charles Wakefield should have seconded it, as well as that you, ladies and gentlemen, should have carried it and received me so very kindly. It is a very great pleasure to me to have at last taken the post of Grand President of the League of Mercy. I must confess to feeling somewhat shy, standing here, when I know that I have not as yet been able to take that very close interest which I am anxious to do in the League. As you know, I have been away for the last four months in Canada; I also paid a short visit to the United States. And I am afraid I am not going to be very long in the old country before I start off on a new journey. But let me assure you that I realise the very great importance of the voluntary hospitals; I know the splendid work they have done in the past, especially during the war, and how more necessary than ever they are now; also that a greater strain than ever before is put upon them owing to the increased cost of living and many other difficulties. So this League has more work than ever to do. I am sure we are all encouraged by the kind letter from His Majesty the King, which I have read to you; and may I, in again thanking you for your kindness, wish all further success and prosperity to the League of Mercy? (Loud applause.)

The meeting then terminated.

